# News and Events of the Week

**Published:** 1910-09-24

**Authors:** 


### News and Events of the Week.

Mr. David Davies, M.P., presided at a conference held at Shrewsbury last week to support a National Memorial in Wales to King Edward in the form of a sanatorium for consumptives. The memorial is estimated to cost £300,000, and Mr. Davies has already received promises for nearly £150,000. The Montgomery County Council yesterday agreed to support the scheme.

Dr. W. J. Simpson, Professor of Hygiene at King's College and Lecturer at the London School of Tropical Medicine, has suggested to the Lord Mayor as a King Edward Memorial an Imperial Institute of Hygiene and Tropical Medicine to be devoted primarily to the scientific study of the causes and prevention of endemic and epidemic diseases affecting the Empire.

All previous records were beaten by the recent hospital parade held at Portslade, near Brighton, which succeeded in raising over £60, of which the Brighton and Hove Dispensary received nineteen guineas, and the Sussex County Hospital ten guineas. As the weather was bad on the day of the Parade, this success is all the more creditable to the Committee and to its workers.

Dr. Harrington has completed his description of the etched work of Sir Francis Seymour Haden. The title of the book will be "The Engraved Work of Sir Francis Seymour Haden, P.R.E. : an Illustrated and Descriptive Catalogue," and it will be issued in a handsome quarto volume, the edition of which will be very limited. It will be illustrated with 250 plates, and will be published by Messrs. Henry Young and Sons, of Liverpool.

A new medical body, known as the Medical Library Association, has been formed to bring together those engaged or interested in medical libraries and medical literature. The association intends to maintain an exchange for the distribution of duplicate books, to increase existing facilities for reference work, and to encourage the study of the history of medicine. Meetings have been held at South Kensington, at which Professor Osler has presided, and papers on medical library work have been read by the librarians of the Royal Colleges of Physicians and Surgeons and the Royal Society of Medicine of London.

It is a truism to state that the average visitor to London from abroad knows more about the history of the capital and has seen more of its historic buildings than the great majority of its millions of citizens, and yet this ignorance on the part of Londoners must be diminishing in the face of the enticing books about London which are now beginning to appear at popular prices. One of these is shortly to be issued by Messrs. A. & C. Black under the simple title of "London." It is to be illustrated in colour with numerous reproductions of the work of Mr. Herbert Marshall, R.W.S., Mr. W. L. Wyllie, R.A., Mr. Philip Norman, and others. The text, by Mr. A. R. Hope Moncrieff, is a very readable medley of description, history, reminiscence, and anecdote, and its perusal should add much romance as well as interest to those whose lives are spent in the teeming ways of London.

Sir Shirley Murphy, Medical Officer of Health of the County of London, has been added to the Mansion House Committee of the King Edward Memorial Fund. Lady Douglas, writing to the Lord Mayor on the form of the memorial, suggests a sanatorium for soldiers who are invalided out of the Army in an advanced state of consumption.

Mr. Whitehead, the Vaccination Officer of the Haslingden Union, has asked for a revision of his rate of pay, pointing out that, owing to conscientious objections, the number of certificates had fallen in five years by eight hundred and his salary by nearly £30 a year. The Guardians declined to increase the rate of pay, and Mr. Whitehead placed his case before the Local Government Board. The Board requested the Guardians to make some revision, but they have refused.

The Hull Medical Officer of Health's report for 1909 states that the total number of practising midwives in Hull (forty-five) shows a decrease of seven from the preceding year and of thirteen from the year 1905; and it states that "though no shortage of midwives occurs at present, the decrease points to the necessity of some provision being made for the training of suitable women." "The cost of training, however," it is added, "appears to be a barrier to many who would discharge the duties both efficiently and well." Of the forty-five there are twelve hospital-trained certified midwives and thirty-three *bonâ-fide* (untrained certificated) midwives.

As a result of the Vaccination Act of 1907 the fees of Mr. H. M. Collins, Vaccination Officer to the Hammersmith Guardians, fell off, and an application to the board for increased remuneration the Guardians refused on the ground that previously he had his fees increased on his promising to do additional work, which he now did not and would not do. Mr. Collins then laid his case before the Local Government Board, and after some correspondence the Assistant Secretary to the Local Government Board stated to the Guardians that in the Board's opinion Mr. Collins deserved compensation in respect of this loss. The Guardians have decided not to amend their previous decision. Mr. Collins is waiting to see what steps the Local Government Board will take in face of this attitude.

The existence of a naval Power in the Mediterranean far older than even classic Greece is a highly interesting discovery for British people, and the story of the sea-kings of Crete revealed by recent discoveries in the island is one of absorbing interest. The announcement, therefore, that Messrs. A. & C. Black are just about to publish a volume from the pen of the Rev. James Baikie, entitled "The Sea-Kings of Crete and the Prehistoric Civilisation of Greece," is one of particular interest. The book is to be amply illustrated with photographs and will be provided with a map of Crete and a plan of the Palace of Knossos, where very extensive remains have been brought to light. Mr. Baikie has written his book with a view to presenting to the general reader in an untechnical and interesting form the astonishing results of the various explorations which have proved the existence of a sea Power.

THE Apollinaris Company has been awarded the Grand Prix at the Brussels Exhibition for its exhibit of water from the famous spring in the Ahr valley.

A PROPOSAL for the Corporation of London to commemorate the services of the late Miss Florence Nightingale, who received the honorary Freedom of the City in 1908, by placing a marble bust of her in the Guildhall, or in some other way, will come before the Corporation at an early date.

THE following are the arrangements for the week at the Medical Graduates' College and Polyclinic, 22 Chenies Street, W.C., at 4 P.M. : Monday, September 26, Dr. Willmott Evans, Clinique (skin); Tuesday, September 27, Dr. Newton Pitt, Clinique (medical); Wednesday, September 28, Mr. M. P. Mayo Collier, Clinique (surgical); Thursday, September 29, Dr. F. J. Poynton, Clinique (medical); and Friday, September 30, Mr. M. S. Mayou, Clinique (eye).

AT Teignmouth, on September 8, an inquest was held on May Lear, of Tamworth, Staffordshire, who died after taking a headache powder. Evidence was given to the effect that on the morning previous to her death the woman had taken a headache powder. While out walking with her husband she complained of feeling ill, and a doctor was sent for, but the woman died almost immediately. The doctor, replying to the Coroner, stated that the husband had handed him one of the powders which his wife was in the habit of taking for headache. It was a powder containing acetanilide, a powerful cardiac depressant. These powders were sold without any restriction, and the drug was about the most dangerous that could be given for headache.

THE Selborne Society has been investigating the flora of a building site in Farringdon Street, scarcely removed from the heart of the City. Although this plot has only been cleared for about two years, 28 species of flowering plants and ferns have established themselves upon it. Mosses, liverworts, and others of the more simple plants are also represented. Mr. J. C. Shenstone, F.L.S., is preparing a detailed list which will be published in the October number of the *Selborne Magazine*. In a preliminary report he says that one would expect "wind distributed plants" to figure largely in this garden, but not bracken, which is a difficult fern to transplant.

Two cases of cerebro-spinal fever have been notified in the parish of Sandon, near Chelmsford, and one has proved fatal. The cases were in adjoining blocks of cottages, and there have probably been other cases, as several children had suffered from headache, sickness, etc., early signs of the disease; but only the two mentioned exhibited typical symptoms. Every possible precaution has been taken, and Dr. Thresh, the county medical officer, states that he had expected something of the sort in Essex, in which he has had four small outbreaks since he has been the county officer, but in neither of these instances was there any spread. The disease has also broken out afresh in Nottingham, after a lapse of nearly three weeks. The Corporation Hospital at Bagthorpe is now entirely free from patients suffering from the disease, and the cases under treatment in the county area are pursuing a normal course. The visit of Dr. Farrar, the medical officer deputed by the Local Government Board to institute special inquiries in the locality, and his statements regarding the outbreaks, have done much to allay public anxiety.

THE annual dinner of the past and present students of the Middlesex Hospital Medical School and their friends will take place at the Trocadero, Piccadilly Circus, on Monday, October 3, at 6.30 for 7 o'clock precisely, Thomas H. Kellock, Esq., M.A., M.D., M.C., F.R.C.S., in the chair.

THE death of a prisoner, George Warner, aged twenty-eight, from enteric in Wandsworth Prison, formed the subject of an inquiry by Mr. John Troutbeck, the District Coroner, at the prison on Tuesday last. Warner was undergoing a sentence of twelve months for shopbreaking. On his reception in May he was certified as fit for hard labour. Dr. J. J. Pitcairne, the principal medical officer, said that Warner complained of illness on July 13 and was admitted to hospital. At first the case was by no means a typical one. He was of opinion that the attack must have originated in the prison. It was practically impossible that it could have originated outside. There was a doubtful case early this year, but before that there had been nothing for two years. The drainage system was now as nearly perfect as modern science could make it, and there was no possible contamination from the water-supply. The jury returned a verdict of "Natural death from enteric fever."

